# Pulmonary Disease Associated with Nontuberculous Mycobacteria, Oregon, USA

**DOI:** 10.3201/eid1709.101929

**Published:** 2011-09

**Authors:** Kevin L. Winthrop, Cara D. Varley, Jill Ory, P. Maureen Cassidy, Katrina Hedberg

**Affiliations:** Author affiliations: Oregon Health and Sciences University, Portland, Oregon, USA (K.L. Winthrop, C.D. Varley);; Oregon Public Health Division, Portland (K.L. Winthrop, P.M. Cassidy, K. Hedberg);; Ory Consulting, Portland (J. Ory)

**Keywords:** nontuberculous Mycobacteria, tuberculosis and other mycobacteria, prevalence, epidemiology, pulmonary disease, spatial distribution, geographic information system, Oregon, United States, letter

**To the Editor:** Nontuberculous mycobacteria (NTM) are environmental organisms ubiquitous in soil and water, including municipal water supplies. When inhaled, these organisms cause chronic, severe lung disease in susceptible persons (*1*). Recent epidemiologic studies suggest NTM pulmonary disease is increasingly prevalent in North America, with annual incidence rates of 13 cases per 100,000 population in persons >50 years of age and 2–4-fold higher in older age groups (*2**–**4*). The current distribution of pulmonary NTM disease has been poorly characterized with regard to environment, climate, and other factors.

We recently performed a statewide NTM surveillance project in Oregon, United States, where we documented higher pulmonary disease rates within the moister, temperate western regions of the state. Oregon is bisected north-south by mountains into 2 distinct climate zones. Western Oregon, where 87% of the state’s population lives, is temperate and wet; eastern Oregon is primarily rural, with an arid, high desert climate. Our goal was to evaluate whether disease clustering within the state could be explained by population density.

For all Oregon residents who had newly diagnosed and existing pulmonary NTM disease during 2005 and 2006, we used case-patient home ZIP code and county of residence to construct statewide disease maps (*4*). We obtained state ZIP code and county-level census data for 2005 and 2006 from the Portland State University Population Research Center and used Oregon Office of Rural Health criteria to designate ZIP codes as urban or rural and counties as rural (nonmetropolitan), micropolitan, or metropolitan (*5**,**6*). Unlike ZIP code data, which lacked age information, county census data were age stratified and consisted of population numbers aggregated in 5-year age groups (e.g., 0–4 years, 5–9 years). Because nearly all pulmonary NTM disease occurred in persons >50 years of age, we calculated age-adjusted disease prevalence rates (by using 95% Poisson exact confidence intervals) for patients >50 years of age in the county census data. We used the Cochran-Armitage test for trend to evaluate differences in rates by rural, micropolitan, and metropolitan county designations.

Statewide, 385 (94%) of 411 NTM cases occurred among residents of western Oregon, and the crude rate of annual disease prevalence was significantly higher in western than in eastern Oregon (6.0 vs. 2.7/100,000; p<0.05) (Figure A1). Within the western region, rates were significantly higher in urban than in rural ZIP codes. Using county-level data, we found that age-adjusted prevalence rates in western Oregon strongly correlated with increasing levels of population density (Table). In eastern Oregon, where only 26 cases occurred, age-adjusted rates among residents >50 years of age were similar (7.6 cases/100,000 population) to those in rural counties within western Oregon (6.5/100,000).

**Table:** Prevalence of pulmonary nontuberculous mycobacterial disease, by geographic region and population density, Oregon, USA, 2005 and 2006*

Region/population density		Prevalence† (95% confidence interval)		
	Total no. cases	All age groups	Age <50 y	Age >50 y
Total	411	5.6 (5.0–6.1)	1.1 (0.8–1.4)	15.2 (13.6–16.9)
County-level analysis				
Western	385	6.0 (5.4–6.6)	1.2 (0.9–1.6)	16.5 (14.8–18.4)
Metropolitan	341	6.3 (5.7–7.0)	1.3 (1.0–1.8)‡	18.0 (16.0–20.2)§¶#
Micropolitan	39	4.5 (3.2–6.1)	0.2 (0.0–1.0)‡	11.1 (7.8–15.2)§¶
Rural	5	3.6 (1.2–8.3)	1.3 (0.0–7.1)	6.5 (1.8–15.9)§#
Eastern	26	2.7 (1.8–4.0)	0.2 (0.0–0.9)	7.6 (4.9–11.2)
Metropolitan	9	3.0 (1.4–5.6)	0.0 (0.0–1.8)	8.6 (3.9–16.3)
Micropolitan	12	2.5 (1.3–4.3)	0.3 (0.0–1.7)	6.8 (3.4–12.2)
Rural	5	2.9 (1.0–6.8)	0.0 (0.0–3.5)	7.8 (2.5–18.2)
ZIP code**				
Western	365	5.7 (5.2–6.4)	–	–
Urban	277	6.4 (5.7–7.2)††	–	–
Rural	88	4.3 (3.5–5.3)††	–	–
Eastern	26	2.8 (1.8–4.0)	–	–
Urban	5	2.7 (0.9–5.7)	–	–
Rural	21	2.8 (1.7–4.1)	–	–

*Annualized 2-year disease period prevalence, Oregon, 2005 and 2006. †Cases per 100,000 population. ‡Significant differences (p<0.05) between western Oregon metropolitan and micropolitan counties, age <50 y. §West, age >50 y, metropolitan, micropolitan, and rural (Cochran-Armitage test for trend, p<0.01). ¶Significant differences (p<0.05) between western Oregon metropolitan and micropolitan counties, age >50 y. #Significant differences (p<0.05) between western Oregon metropolitan and rural counties, age >50 y. **Included only the 391 patients for whom with ZIP code data were available. –, age-level data not available for ZIP codes. ††Significant differences (p<0.05) between western Oregon urban and rural ZIP codes.

In Oregon, where most pulmonary NTM disease is caused by *Mycobacterium avium* complex (MAC), our findings suggest that the higher rates of disease in the wet western portion of the state are best explained by differences in population density (*4*). Disease rates there were highly correlated with increasing population density, and in rural areas of western Oregon, disease rates were similar to those in the arid, primarily rural eastern portion of the state.

Humans presumably are exposed to NTM daily through showering, bathing, and other activities where water or soil is aerosolized (*7*). Previous environmental studies suggest that persons living in urban areas could potentially have greater NTM exposure during these activities because NTM is more prevalent in piped networks of municipal water systems than in well-water systems primarily used in rural regions (*8*). A study in Japan in the 1980s found a similar association of pulmonary NTM disease (primarily MAC) with urban and wet environments compared with arid and rural regions in our study but unlike our study was not able to evaluate differences in disease rates between urban and rural areas independent of climate differences (*9*). A 1979 Texas study found an association of pulmonary NTM with rural living, although this result was driven by *M. kansasii* disease, and rates of MAC were actually higher in rural areas (*10*). These and other similar studies were conducted decades ago when the epidemiology of NTM was substantially different (i.e., predominantly a disease of male patients) and before the formulation of the 2007 American Thoracic Society/Infectious Diseases Society of America pulmonary NTM disease criteria (*1*).

We were limited in drawing firm conclusions about why pulmonary NTM is more common in urban areas because we were not able to evaluate patients or regional water systems within our study. Persons living rurally might be less likely to seek medical care and thus have NTM diagnosed, which would account for the differences in our study. However, given the reasonably close proximity of western Oregon’s rural regions to major medical centers, we believe this scenario is unlikely.

Our findings suggest that pulmonary NTM disease is closely associated with urban living. We suspect the difference in disease rates between urban and rural areas might reflect differences in host exposure to these pathogens. Further studies should be undertaken to elucidate the environmental exposures associated with pulmonary NTM.

## Figures and Tables

**Figure A1:**
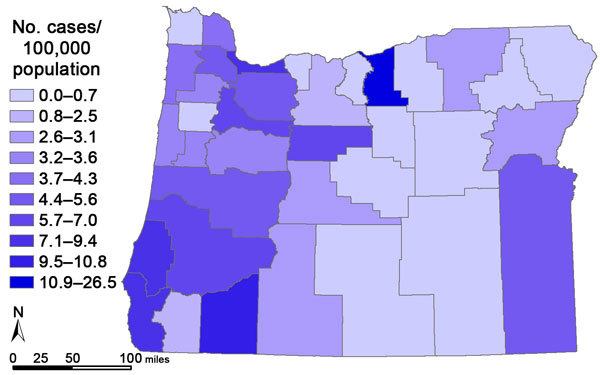
Rates of pulmonary nontuberculous mycobacterial disease, by county, Oregon, USA, 2005 and 2006.
